# Intrathecal Injection of Amitriptyline and Doxepin for Spinal Anesthesia in Animal Studies

**DOI:** 10.5812/kowsar.22287523.2127

**Published:** 2011-09-26

**Authors:** G. Ulufer Sivrikaya

**Affiliations:** 1 Department of Second Anesthesiology and Reanimation, Sisli Etfal Training and Research Hospital, Istanbul, Turkey

**Keywords:** Spinal anesthesia, Amitriptyline, Doxepin

*Dear Editor*,

I read with a great interest the recent article by Alebouyeh et al. about intrathecal administration of amitriptyline and doxepin to induce spinal blockade and comparison of their effects with those of bupivacaine (1).

Alebouyeh et al. reported that intrathecal injection of various drugs with different mechanisms is an intriguing issue in anesthesiology and pain management. Antidepressants are complex drugs having multiple actions, including effects on serotoninergic, adrenergic, and opioid mechanisms, and may modulate the levels of various neurotransmitters (gamma-amino butyric acid [GABA], adenosine, N-methyl D-aspartate [NMDA], and cholecystokinin) and ion channels (Na+, K+, Ca++) (2). Thus, the different of pharmacological actions of antidepressants contributes to their efficacy in relieving depression at central sites, in producing analgesia at supraspinal, spinal, and peripheral sites, as well as in the side effects observed at multiple sites.

Antidepressants belonging to a same class also show difference in the intensities of pharmacological actions, some accounting for differential actions and side effects, similar to the findings of amitryptyline and doxepin reported by Alebouyeh et al. Tricyclic antidepressants (TCAs) have very different efficacy as local anesthetics in vivo (3). Among various TCAs, amitriptyline and doxepin have been studied as local anesthetics in most studies. Chen YW et al. evaluated the spinal anesthetic effect of various TCAs after a single intrathecal injection (4). In this study, the effects of 9 TCAs, including amitriptyline and doxepin, and 3 traditional local anesthetics (bupivacaine, lidocaine, and mepivacaine) were evaluated in rats. The results showed that intrathecal amitriptyline had a more potent and longer duration of spinal anesthetic effect than bupivacaine (under a given concentration of 5 mM), whereas the potency of other TCAs was similar to or less than that of bupivacaine.

Although meta-analyses on the maximum allowable dose for intrathecal injection of these drugs have not been performed, several studies have reported the administration of these drugs in different doses. The study performed by Alebouyeh et al. has contributed to these findings; In a study performed by Cerda et al. , intrathecal injection of varying doses of amitriptyline (0. 25, 1, and 5 mg) in sheep using low thoracic catheters did not significantly affect spinal cord blood flow or hemodynamic variables (5). In another study, amitriptyline was administered intrathecally at doses of 25, 50, and 100 μg/rat to investigate the effect on carrageenan-induced paw edema in rats and no side effects were observed (6). Sudoh et al. used a route of administration different from intrathecal administration; they administered amitryptyline and doxepin 5 mM as a single injection via the sciatic notch of rats (3) and administered doxepin 2. 5, 5. 0, and 10 mM in a rat sciatic nerve model (7). In the latter case, possible morphological alterations were assessed by light microscopic examination in the cross-sections of the sciatic nerves; there was no evidence of neurotoxicity, and they concluded that doxepin seemed to be nontoxic to peripheral nerves at concentrations up to 10 mM.

However, a number of studies have shown concentration-dependent toxicity when these drugs are applied to the peripheral nerve or are administered intrathecally (8-10). Safety is an important aspect of intrathecal administration of novel agents; safety of administration can be evaluated by determining not only the potential effect on function (e. g. , motor, sensory, autonomic) but also changes in spinal morphology. In another study by Sudoh et al. , spinal blockade after intrathecal administration of various concentrations of amitriptyline in a fixed high volume and histopathologic changes in the spinal cord were evaluated (9). They administered 100 µL of 5, 10, 15. 9 (0. 5%), 25, 50, or 100 mmol/L amitriptyline hydrochloride solution intrathecally to rats. At doses of 50 and 100 mmol/L, many rats did not completely recover from spinal block. In addition, severe histopathologic changes were seen in the spinal root section of animals injected with concentrations higher than 25 mmol/L amitriptyline. Intrathecal administration of amitriptyline at high doses is not recommended because it might cause irreversible neurologic deficit. In a study by Gerner et al. , 60 µL of doxepin at 10, 20, and 50 mM was injected through intrathecal catheters implanted in the lumbar region of rats (11). Their results showed that intrathecal doxepin most likely has a very narrow therapeutic range approximately 10–20 mM for complete blockade and higher concentrations are likely to be neurotoxic. In this study, neurotoxicity, defined as persistent neurological deficit, was observed at 50 mM.

The adverse-effects of antidepressants are primarily related to their pharmacological properties. Some antidepressants have cardiotoxic side effects such as blockade of alpha 1-noradrenergic receptors, which lead to orthostatic hypotension and tachycardia. TCAs also have type 1 antiarrhythmic action and are potent inhibitors of sodium channels (2). Doxepin has lesser cardiotoxicity than other TCAs (11). I agree with the findings reported by Alebouyeh et al. that cardiotoxicity might be the most likely reason of deaths.

Although TCAs, mainly amitriptyline, followed by doxepin, appear to be more effective than bupivacaine in inducing spinal blockade, their poor tolerability profile (discussed below) and need for careful dose titration (because of a low therapeutic index) are major limitations of administration of these drugs.

In conclusion, more preclinical toxicity studies are necessary before introducing intrathecal amitriptyline and doxepin for use in humans. Studies to assess the safety and the therapeutic range of intrathecal application of these drugs will be very useful clinically.
